# Comparative Analysis of Classic and Novel Antitussives on Cough Suppression in Guinea Pigs

**DOI:** 10.3390/pharmaceutics17111423

**Published:** 2025-11-03

**Authors:** Paula Montero, Inés Roger, Erika Esposito, Javier Milara, Julio Cortijo

**Affiliations:** 1 Department of Pharmacology, Faculty of Medicine, University of Valencia, 46010 Valencia, Spain; irola3@gmail.com (I.R.); xmilara@hotmail.com (J.M.); julio.cortijo@uv.es (J.C.); 2 Biomedical Research Networking Centre on Respiratory Diseases (CIBERES), Health Institute Carlos III, 28029 Madrid, Spain; 3 Fundación de Investigación Hospital General Universitario de Valencia (FIHGUV), 46014 Valencia, Spain; 4 Faculty of Health Sciences, Universidad Europea de Valencia, 46010 Valencia, Spain; 5 Department of Pharmacy, School of Medicine and Surgery, University of Napoli Federico II, 80141 Napoli, Italy; erika.esposito3@unina.it; 6 Pharmacy Unit, University General Hospital Consortium, 46014 Valencia, Spain

**Keywords:** antitussive agents, acute cough, guinea pig model

## Abstract

**Introduction:** Cough is a common reflex that serves a protective role, but when persistent, it can severely affect patients’ quality of life. Despite its high prevalence, current treatment options remain limited. Traditional antitussives such as codeine and dextromethorphan present notable side effects, underscoring the need for safer and more effective alternatives. **Methods:** This study evaluated and compared the antitussive efficacy of classic (codeine, cloperastine, dextromethorphan, levodropropizine) and novel (gefapixant) agents using a citric acid-induced cough model in guinea pigs. Animals were pretreated with the selected compounds, and cough frequency, latency to first cough, and cough intensity were assessed using acoustic recordings and quantitative analysis. **Results:** Codeine, cloperastine, and gefapixant produced a significant reduction in cough frequency and markedly increased latency to the first cough, with comparable efficacy at the highest doses tested. In contrast, dextromethorphan and levodropropizine did not significantly affect these parameters. Additionally, cloperastine, codeine, and gefapixant reduced cough intensity, while none of the treatments significantly altered cough duration. **Conclusions:** In this preclinical model, cloperastine and gefapixant demonstrated antitussive effects comparable to codeine but without the known narcotic-associated risks. These findings highlight the potential clinical relevance of both centrally and peripherally acting non-opioid alternatives. Continued investigation of these agents may help address the unmet need for safer and more effective cough treatments.

## 1. Introduction

Cough is a physiological reflex that plays a defensive role in clearing infectious agents, foreign particles, or excessive secretions from the respiratory tract. Depending on its duration, cough can be classified as acute, when it lasts less than four weeks, or chronic, when it persists for more than eight weeks [1]. In terms of characteristics cough may be productive, involving expectoration of mucus, or dry, such as irritative [2,3]. Importantly, most patients with chronic cough present with a dry or minimally productive cough [4]. Usually, dry or irritative cough has limited clinical usefulness. Instead, it becomes a bothersome reflex, often worsening patient discomfort without providing physiological benefit. Cough can arise as a secondary symptom in a variety of pulmonary conditions, including asthma, chronic obstructive pulmonary disease, idiopathic pulmonary fibrosis, and lung cancer [5]. It is also a common feature of upper respiratory tract infections, and acute episodes may be triggered by exposure to irritants or allergens [1].

Acute cough affects a significant portion of the population—up to 64% at any given time [6]. Consequently, it can have a substantial impact on patients’ quality of life [7]. It may lead to sleep disturbances, fatigue, urinary stress incontinence, and work absenteeism. Additionally, it can cause social embarrassment, as patients may experience social withdrawal due to the persistent nature of the symptom. In more severe instances, complications such as rib fractures, pneumothorax, pneumomediastinum, and subcutaneous emphysema can occur [4]. Given this level of discomfort and disruption, it is not surprising that most individuals actively seek medical treatment [8]. However, to date, more effective therapies are needed to treat chronic cough [9].

Despite its prevalence and the burden it imposes, no new pharmacological treatments for acute cough have been approved in over three decades [10]. Although some drugs, like cloperastine, remain relevant in countries such as Spain due to their established safety and efficacy profiles.

Current treatment options for chronic cough primarily include centrally acting antitussives such as codeine and dextromethorphan. However, their clinical utility is limited due to a range of adverse effects. The adverse effects include sedation, potential for abuse, and risk of overdose [11]. Codeine is associated with respiratory depression, a serious concern especially in vulnerable populations, and can also lead to constipation. On the other hand, dextromethorphan causes neurological and gastrointestinal side effects. Common adverse reactions include drowsiness, dizziness, fatigue, vertigo, as well as digestive discomfort [12].

These limitations underscore the need for novel therapies that modulate cough reflex sensitivity without engaging central nervous system pathways [13]. In this context, the guinea pig model is as a reliable preclinical tool for evaluating the efficacy of antitussive agents. The present study aims to provide updated comparative evidence on the efficacy of both established and novel antitussives. In addition to traditional agents such as codeine and dextromethorphan, this study examines cloperastine—a centrally acting compound with antihistaminic and papaverine-like properties but devoid of narcotic effects [14]—and levodropropizine, a peripherally acting non-opioid agent that suppresses cough by modulating the release of sensory neuropeptides [15]. Furthermore, we assess the antitussive potential of gefapixant, a P2X3 receptor antagonist representing a novel therapeutic class with peripheral selectivity [16]. This investigation is the first to conduct a direct comparative analysis of these diverse agents, including both conventional and emerging therapies, to better inform future treatment strategies for cough.

## 2. Materials and Methods

### 2.1. Animals

Dunkin Hartley guinea pigs of both sexes, weighing between 200 and 250 g, were obtained from Charles River Laboratories (L’Arbresle, France). Males and females were distributed as evenly as possible across groups; in cases where the number of animals was odd, one additional male was included. Upon arrival, animals were quarantined following institutional protocols and housed under standard laboratory conditions with ad libitum access to food and water. Prior to the experimental procedures, guinea pigs were weighed and randomly assigned to the different treatment groups (N = 3–8 animals per group). These included a negative control group receiving 0.9% saline solution (B. Braun Medical S.A., Rubí, Spain) (*n* = 4), a positive control group exposed to 0.4 M citric acid (Sigma-Aldrich, St. Louis, MO, USA) (*n* = 8), and various drug treatment groups. The drugs administered were cloperastine at doses of 6 mg/kg (*n* = 4), 12 mg/kg (*n* = 4), and 24 mg/kg (*n* = 7); codeine at 6 mg/kg (*n* = 5), 12 mg/kg (*n* = 6), and 24 mg/kg (*n* = 7); dextromethorphan at 32 mg/kg (*n* = 6); levodropropizine at 72 mg/kg (*n* = 4); and gefapixant at 6 mg/kg (*n* = 3), 12 mg/kg (*n* = 4), and 24 mg/kg (*n* = 3). All drugs were purchased from Selleckchem (Planegg, Germany). The selected doses for each drug were established based on previously published data [14,17,18,19,20,21,22,23,24]. All treatments were administered orally by gavage 30 min before citric acid exposure, using a vehicle composed of 0.9% saline supplemented with 0.6% Methocel and 1.5% PEG400 (Sigma-Aldrich, St. Louis, MO, USA). At the end of the procedure, animals were euthanized with an intraperitoneal injection of sodium pentobarbital(Baxter S.L., Madrid, Spain). All animal procedures were approved by the Institutional Animal Ethics Committee of the University of Valencia (Approval number EVRTE/2024/1877794; Facility Code: ES460780001001; Approval date: 28 May 2024).

### 2.2. Cough Induction Protocol and Recording Analysis

To evaluate the cough response, unrestrained guinea pigs were individually placed inside a transparent chamber with a volume of 2500 cm^3^. Animals were then exposed to a continuous aerosol of either 0.9% saline or 0.4 M citric acid, dissolved in 0.9% saline, for a period of 7 min. Aerosols were generated using an ultrasonic nebulizer (Omron NE-U780, Omron Healthcare Europe B.V., Hoofddorp, The Netherlands) with a nebulization rate of 3 mL/min and an aerosol output rate of 0.14 mL/min. Following nebulization, animals remained in the chamber for an additional 7 min to allow for continued observation. The total number of coughs was manually recorded by continuously monitoring the animals, taking into account both the sound characteristics and the behavioral signs that distinguish coughs from sneezes. Acoustic data were captured using an omnidirectional electret microphone (Monacor ECM 3005, frequency response 50–16,000 Hz) (Monacor International GmbH & Co. KG, Bremen, Germany), which was placed inside the chamber and connected to a computer for simultaneous audio recording. All experiments were conducted in an environment with minimal background noise to ensure accurate sound capture.

### 2.3. Cough Quantification and Acoustic Data Analysis

Cough events were manually counted in real-time during each experiment by two trained observers positioned outside the exposure chamber. The observers continuously monitored each animal for a total of 14 min, recording both the number and onset time of each cough. Observers were blinded to the experimental groups to minimize bias in cough identification and counting. Cough identification was based on characteristic behavioral and acoustic patterns, and every event was categorized as either (i) confirmed cough by both observers, (ii) confirmed by one observer but not the other, or (iii) a different behavior such as sneezing, confirmed by both as not being a cough. This initial manual count was subsequently validated through visual inspection of the spectrograms generated from the audio recordings to ensure accurate identification of cough events.

To complement the manual analysis and provide an objective quantification of the cough response, audio data were further analyzed using Audacity software (version 3.6.4, https://audacityteam.org/). To minimize background noise, the noise reduction tools integrated in Audacity were applied as needed to further clarify cough events. From these filtered recordings, several acoustic parameters were extracted. Recordings were examined through both auditory review and visual spectrogram analysis to identify individual cough events with greater precision and to count the total number of coughs. Additionally, the parameters extracted with the software included the onset time of the first cough and the duration of each individual cough event. To assess intensity parameters, two measurements were used. First, the power spectral density (PSD) distribution was calculated. The PSD was obtained by applying a Fast Fourier Transform (FFT) with a Hanning window to determine the distribution of signal power across frequencies. The resulting spectra represented the power content (dB/Hz) as a function of frequency. Based on previous studies indicating a PSD peak in guinea pig coughs around 1.5 kHz, the analysis focused on the 750–3750 Hz frequency range in order to facilitate between-group comparisons centered around this peak [25,26,27].

Second, the root mean square (RMS) amplitude of each cough signal was calculated, a standard measure that correlates with the acoustic energy of the sound. RMS levels were expressed in decibels relative to the maximum possible value (0 dB), with values closer to 0 indicating more intense signals.

### 2.4. Statistical Analysis

Data were analyzed using GraphPad Prism (GraphPad Software, version 10, San Diego, CA, USA). Because some experimental groups had small sample sizes (*n* = 3–8) and normality could not be reliably assessed, a nonparametric approach was applied. Differences among treatment groups were evaluated using the Kruskal–Wallis test, followed by Dunn’s multiple comparisons test to compare each treatment with the positive control group (citric acid). Results are presented as median ± interquartile range (IQR). A *p*-value < 0.05 was considered statistically significant.

For the power spectral density (PSD) analysis of cough sounds, independent comparisons between the positive control and each treatment group were conducted at each selected frequency point using the nonparametric Mann–Whitney test. Results are presented as mean ± SEM. A *p*-value < 0.05 was considered statistically significant.

## 3. Results

### 3.1. Evaluation of Antitussive Effects

**Figure 1:**
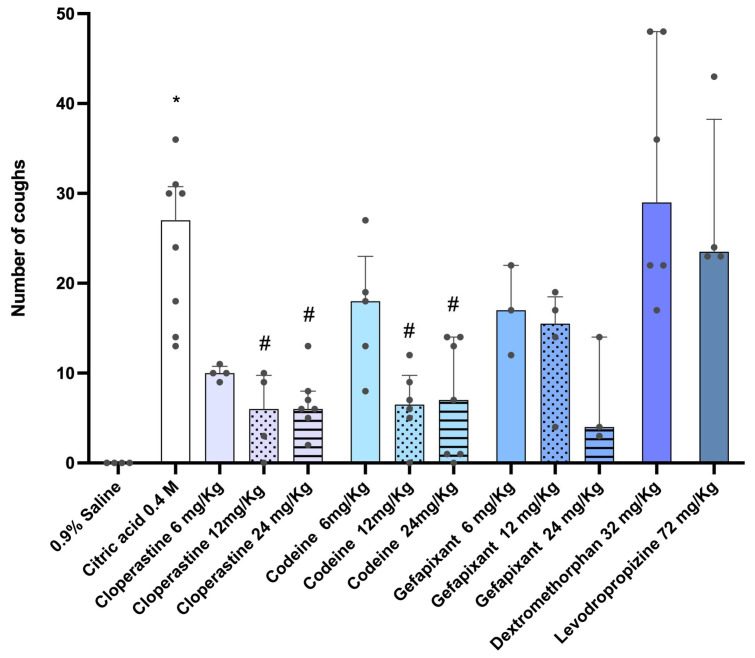
Citric acid aerosol-induced cough responses in guinea pigs. Guinea pigs were orally treated with cloperastine (6, 12, or 24 mg/kg), codeine (6, 12, or 24 mg/kg), gefapixant (6, 12, or 24 mg/kg), levodropropizine (72 mg/kg), or dextromethorphan (32 mg/kg) 30 min before exposure to 0.4 M citric acid aerosol. Coughs were counted in real time for 14 min by two blinded observers and validated by spectrogram inspection using Audacity software. For statistical analysis, nonparametric tests were applied: Kruskal–Wallis followed by Dunn’s multiple comparisons test. Results are expressed as median ± interquartile range (IQR). * *p* < 0.05 vs. 0.9% saline. # *p* < 0.05 vs. 0.4 M citric acid group.

To compare the antitussive effects, the recordings were analyzed to identify individual cough events. As shown in Figure 1, animals exposed to 0.9% saline exhibited no coughing, whereas those exposed to citric acid showed an increase in cough frequency, reaching a mean of 24.5 ± 3 coughs over 14 min. Cloperastine and codeine, at the two highest doses (12 and 24 mg/kg), produced a significant reduction in the number of cough events, achieving approximately a 70% decrease at the highest doses. A similar reduction was observed with gefapixant at the highest dose of 24 mg/kg. However, levodropropizine and dextromethorphan did not produce a notable effect, as they failed to reduce the number of citric acid-induced coughs.

**Figure 2:**
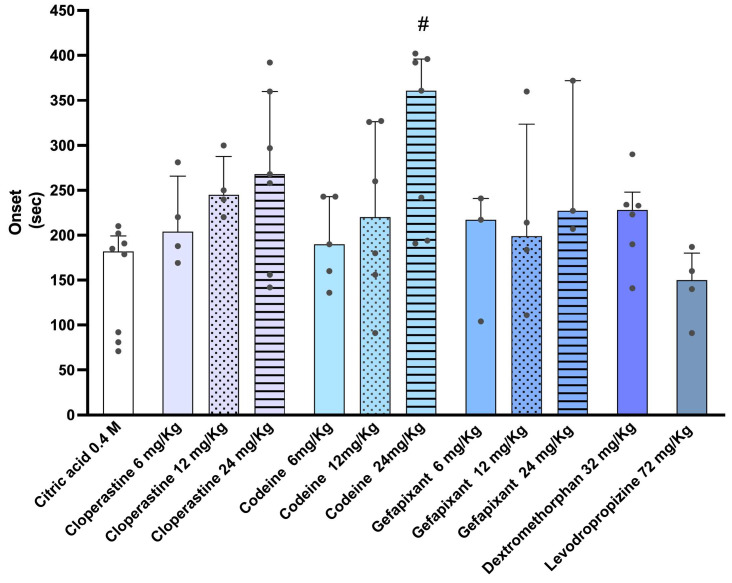
Onset time of citric acid aerosol-induced cough in guinea pigs. Guinea pigs were orally treated with cloperastine (6, 12, or 24 mg/kg), codeine (6, 12, or 24 mg/kg), gefapixant (6, 12, or 24 mg/kg), levodropropizine (72 mg/kg), or dextromethorphan (32 mg/kg) 30 min before exposure to 0.4 M citric acid aerosol. The latency to the first cough (onset time) was recorded by two blinded observers during the 14-min exposure period and subsequently confirmed through spectrogram analysis of the corresponding audio recordings using Audacity software. For statistical analysis, nonparametric tests were applied: Kruskal–Wallis followed by Dunn’s multiple comparisons test. Results are expressed as median ± interquartile range (IQR). # *p* < 0.05 vs. 0.4 M citric acid group.

Regarding onset time, most treatments, except levodropropizine, tended to delay the onset of coughing compared with the citric acid control group (Figure 2). The mean latency to the first cough in animals exposed to citric acid alone was 151.4 ± 20 s. Among the tested drugs, codeine showed the greatest delay, with a mean latency of 311 ± 36 s, followed by gefapixant (268 ± 51 s) and cloperastine (253 ± 38 s). Dextromethorphan also delayed cough onset, although to a lesser extent (218 ± 20 s). In contrast, levodropropizine did not significantly differ from the citric acid group in terms of latency. Only the highest dose of codeine (24 mg/kg) resulted in a statistically significant increase in cough onset time compared with the citric acid group. In addition, analysis of individual cough events showed no significant differences in the duration of single coughs among treatment groups.

### 3.2. Analysis of Cough Intensity

**Figure 3:**
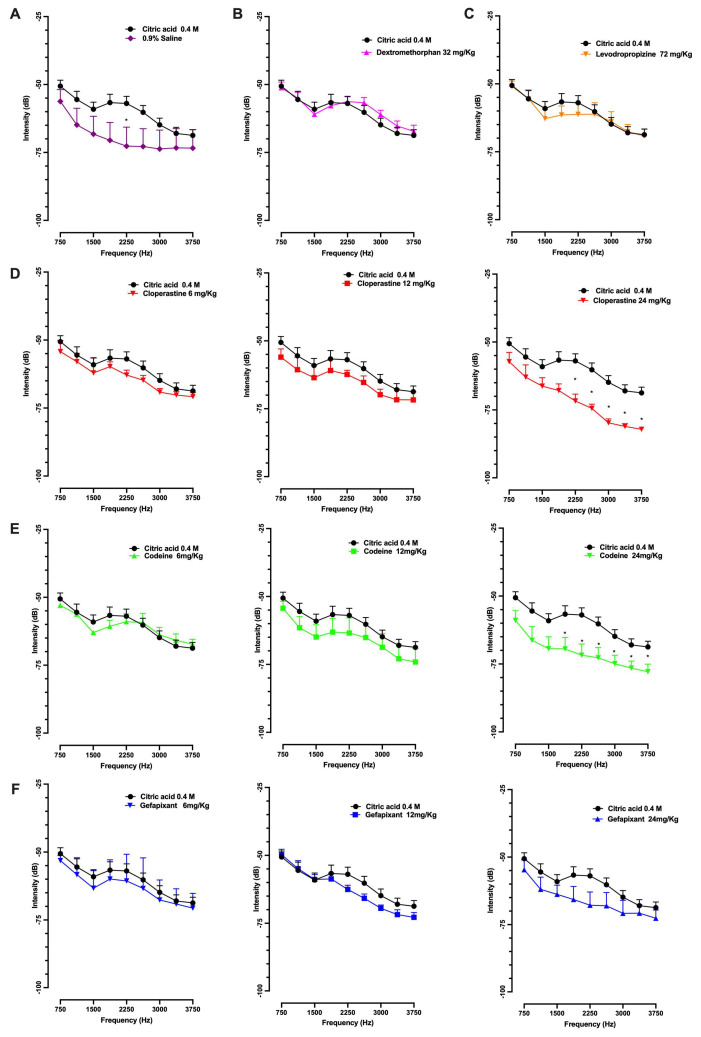
Power spectral density analysis of cough sounds induced by citric acid in guinea pigs. Guinea pigs were orally treated with cloperastine (6, 12, or 24 mg/kg), codeine (6, 12, or 24 mg/kg), gefapixant (6, 12, or 24 mg/kg), levodropropizine (72 mg/kg), or dextromethorphan (32 mg/kg) 30 min before exposure to 0.4 M citric acid aerosol. Cough sounds were recorded throughout the 14-min exposure period and analyzed using Audacity software. The power spectral density (PSD) was examined within the 750–3750 Hz range, and represents sound intensity (dB/Hz) across frequencies. Panels show PSD profiles for each treatment: (**A**) control group (0.9% saline), (**B**) dextromethorphan (32 mg/kg), (**C**) levodropropizine (72 mg/kg), (**D**) cloperastine (6, 12, and 24 mg/kg), (**E**) codeine (6, 12, and 24 mg/kg), and (**F**) gefapixant (6, 12, and 24 mg/kg). For statistical analysis, independent comparisons between the 0.4 M citric acid group and each treatment group were performed at each selected frequency point using a multiple *t*-test with the nonparametric Mann–Whitney approach. Results are expressed as mean ± SEM. * *p* < 0.05 vs. 0.4 M citric acid group.

Regarding cough intensity, two alternative measurements were performed to assess parameter differences within the different groups. Figure 3 displays the power spectral density (PSD) of cough sounds. In Figure 3A, the saline group showed lower intensity values across the analyzed frequency range compared with the citric acid group. For cloperastine, a dose-dependent decrease in cough sound intensity was observed, reaching statistical significance at the highest dose of 24 mg/kg (Figure 3D). Codeine showed a similar trend, with a significant reduction in intensity also detected at the highest dose (24 mg/kg; Figure 3E). Gefapixant followed a comparable pattern, although the changes did not reach statistical significance (Figure 3F). In contrast, dextromethorphan did not produce any detectable decrease in intensity (Figure 3B), while levodropropizine induced a slight but non-significant reduction (Figure 3C).

**Figure 4:**
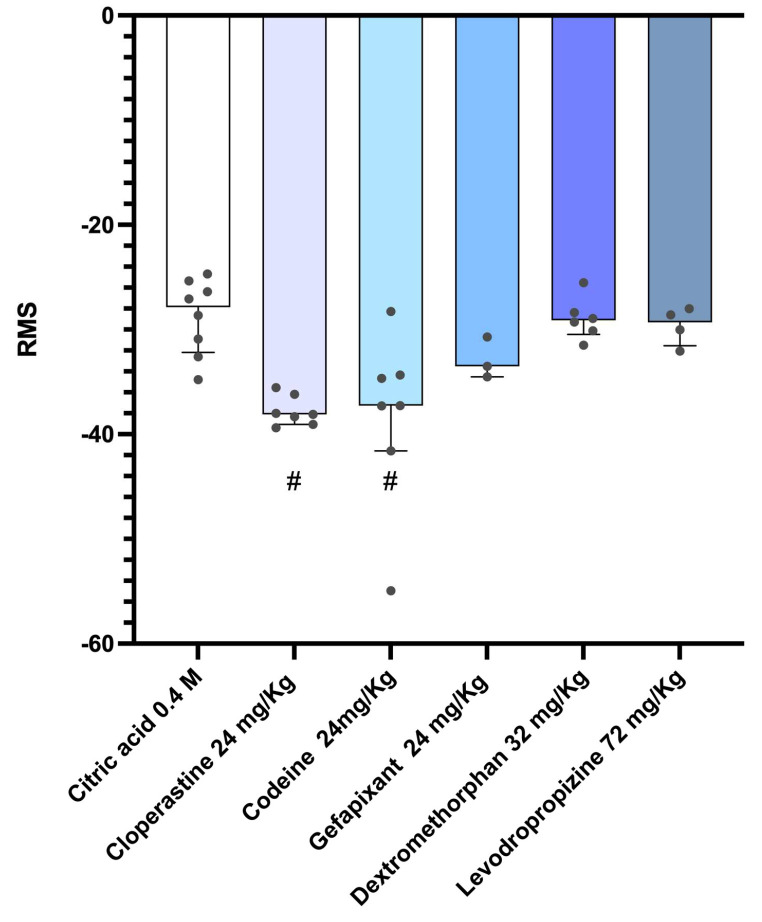
Root Mean Square (RMS) analysis of cough sounds induced by citric acid in guinea pigs. Guinea pigs were orally treated with cloperastine (6, 12, or 24 mg/kg), codeine (6, 12, or 24 mg/kg), gefapixant (6, 12, or 24 mg/kg), levodropropizine (72 mg/kg), or dextromethorphan (32 mg/kg) 30 min before exposure to 0.4 M citric acid aerosol. Cough sounds were recorded throughout the 14-min exposure period and analyzed using Audacity software. The Root Mean Square (RMS) amplitude was calculated to estimate sound intensity, expressed in decibels (dB) relative to the maximum possible value (0 dB), with values closer to 0 indicating more intense cough sounds. For statistical analysis, nonparametric tests were applied: the Kruskal–Wallis test followed by Dunn’s multiple comparisons test. Results are expressed as median ± interquartile range (IQR). # *p* < 0.05 vs. 0.4 M citric acid group.

These findings were further supported by the analysis of the total root mean square (RMS) signal intensity of the cough recordings. In this analysis, higher RMS intensities correspond to values closer to zero, indicating more powerful acoustic signals. As shown in Figure 4, both cloperastine and codeine produced a significant reduction in cough sound intensity at their highest dose (24 mg/kg), consistent with the results obtained from the power spectral density analysis. Gefapixant showed a similar but less pronounced decreasing trend, which did not reach statistical significance. In contrast, dextromethorphan and levodropropizine produced RMS values comparable to those of the citric acid control group, indicating minimal effect on cough intensity.

## 4. Discussion

Cough remains a highly prevalent condition globally, with notable regional variability in both incidence and presentation [28]. Beyond its frequency, cough imposes a considerable social and personal burden, significantly affecting patients’ quality of life [29]. While several antitussive medications are currently available on the market, there is still the need to identify which therapeutic options truly offer meaningful improvements in quality of life [16].

The present study evaluates the efficacy of several antitussive agents using a well-established guinea pig model of citric acid-induced cough. While this model has traditionally relied on manual counting of cough events, advances in audio recording and signal-analysis technologies now enable a more objective characterization of cough, including frequency, latency, and acoustic intensity. Acoustic intensity in particular correlates with airflow velocity and subglottic pressure during the cough reflex, and previous studies have demonstrated positive associations between acoustic energy and physiological markers of cough strength [30,31,32]. Because most preclinical studies compare antitussive activity against placebo or codeine (the historical gold standard), our study included not only codeine, dextromethorphan, levodropropizine, and cloperastine, but also gefapixant, representing a newer generation of antitussive therapies.

When the total cough number was examined over the 14-min observation period, cloperastine and codeine demonstrated comparable efficacy at medium and high doses, and gefapixant also produced a marked reduction in cough frequency at the higher dose. Both cloperastine and codeine significantly prolonged cough latency, further supporting their antitussive effects. Analysis of cough intensity revealed a similar pattern: the highest doses of cloperastine, codeine, and gefapixant produced lower intensity values in the PSD analysis than the citric acid control group. Although the effect of gefapixant did not reach statistical significance, this is likely attributable to the relatively small sample size, which reduced statistical power. Nonetheless, the consistent downward trend suggests a genuine antitussive effect that may reach significance in larger cohorts. RMS intensity results confirmed significant reductions only for high-dose cloperastine and codeine. Minor discrepancies between RMS and PSD outcomes can be explained by their different sensitivities: RMS captures total acoustic energy across the entire recording, whereas PSD reflects the distribution of energy across specific frequencies. Consequently, a treatment may reduce the acoustic energy associated with particular cough-related frequencies, detected as a decrease in PSD, without substantially altering the total sound intensity measured by RMS [31]. Regarding the potential sedative effects of the drugs, no signs of sedation were observed in any treatment group, even at high doses.

The obtained response to codeine, a centrally acting antitussive, was expected, as opioid-based agents remain among the most effective cough suppressants [33]. However, their clinical use is restricted by central nervous system depression, including respiratory depression, which limits long-term safety. This has increased interest in safer, non-opioid alternatives. Our findings indicate that drugs such as cloperastine and gefapixant may achieve antitussive efficacy comparable to codeine without narcotic adverse effects. Cloperastine is the only drug in our panel with dual central and peripheral activity. It acts at the brainstem cough center without depressing respiratory drive, while also targeting receptors in the tracheobronchial tree. Additionally, it has mild bronchodilator and antihistaminic properties [14,22] and is known to inhibit GIRK channels and interact with H1, H3, and sigma-1 receptors, as well as sodium-dependent glucose cotransporters [14,34,35]. Unlike opioid antitussives, cloperastine does not produce respiratory depression or significant sedation, making it a safer therapeutic option [14,36]. Clinical studies have also evaluated the efficacy of cloperastine in patients with cough, supporting its therapeutic relevance. Evidence from clinical trials demonstrates that cloperastine effectively reduces both the frequency and severity of cough in adults and children [14,22,37]. Gefapixant represents a novel pharmacological strategy targeting purinergic pathways. As a selective P2X3 antagonist, it inhibits ATP-mediated activation of airway C-fibers involved in triggering the cough reflex [38]. Clinical trials have shown a 17.6% reduction in cough frequency, although taste-related adverse effects occurred in 69% of patients [38]. Additional phase 3 trials demonstrated significant efficacy in refractory or unexplained chronic cough [39,40], leading to approval in Japan and the European Union [41].

By contrast, levodropropizine and dextromethorphan did not produce noticeable antitussive effects in our model, despite evidence of clinical efficacy in humans [42]. Dextromethorphan, a centrally acting but non-opioid antitussive [43], is not recommended in children [5] and carries abuse potential, as high doses (>1500 mg/day) can induce dissociative effects [44]. Although some citric acid guinea pig studies reported efficacy at similar doses, those used intraperitoneal administration and noted inactivity at lower doses [24]; the greater variability of our oral model suggests that higher doses or larger sample size might have revealed an antitussive effect. Levodropropizine acts peripherally by inhibiting C-fiber activity [33], and a meta-analysis demonstrated significant clinical benefits in adults and children [45]. However, it can cause hypersensitivity reactions such as rash, urticaria, and angioedema [46]. In animal studies it has been effective, but most experiments used cigarette smoke or capsaicin as tussive agents [19,47,48]. Despite their usefulness, cigarette smoke represents only a specific clinical phenotype, typically linked to chronic inflammatory airway diseases, and therefore does not adequately reflect a generalized cough response. By contrast, capsaicin triggers severe bronchoconstriction and dyspnea in guinea pigs, risking loss of consciousness and, consequently, inaccurate and variable cough measurements [49,50]. For these reasons, we selected citric acid as the tussive stimulus, since citric acid-induced cough in conscious guinea pigs is considered the most robust, reliable, and reproducible experimental model available [51]. Given this differential activation, and considering levodropropizine’s peripheral mechanism of action, it is plausible that the drug may be less effective against citric acid-induced stimulation compared with other tussive triggers.

Among the limitations of this study is the relatively small sample size in some experimental groups, which increases variability and consequently reduces statistical significance. Our findings are consistent with previous reports documenting high variability in guinea pig cough studies [52], and several authors have suggested the use of larger sample sizes to improve statistical power. However, this poses an ethical challenge, since animal research guidelines emphasize reducing the number of animals used. Other researchers propose repeated assessments in the same animal to obtain fairly reproducible results with lower variability [53,54], which may represent a suitable refinement for future protocols. In addition, this study aimed to include both male and female animals. Historically, the use of females in preclinical research has often been avoided due to hormonal variability, but we consider it essential to include both sexes to ensure appropriate representation and minimize the translational gap. Previous studies have shown no sex-related differences in the citric acid-induced cough response in guinea pigs [55,56], indicating that there is no justification for excluding females from experimental designs. In our case, the sample size did not allow us to determine whether males and females responded differently to the treatments; however, future studies with larger cohorts should explore whether certain antitussive drugs may be more effective in one sex than the other, especially considering the heterogeneous etiology of cough.

Importantly, the present findings reinforce the value of continuing to search for non-opioid antitussives. Our results indicate that drugs with alternative mechanisms of action, such as cloperastine and gefapixant, may achieve efficacy comparable to codeine while avoiding its adverse effects. For translational purposes, robust in vivo models, such as the guinea pig, remain essential, although larger sample sizes are needed to overcome the intrinsic variability of this model. It should also be emphasized that the mechanisms that initiate cough are heterogeneous [57], and patients with different underlying causes exhibit disease-specific alterations in airway sensory nerve function [58]. Therefore, future studies should aim to identify which cough phenotypes may benefit most from specific therapies, as well as develop peripherally acting drugs that target multiple pathways while minimizing potential adverse effects.

## 5. Conclusions

In summary, this comparative analysis demonstrates that codeine and cloperastine produced the most robust and consistent antitussive effects, while gefapixant showed promising efficacy as a non-opioid alternative. In contrast, the limited activity of dextromethorphan and levodropropizine highlights the heterogeneity of current antitussive therapies and reinforces the need for more selective and mechanism-based approaches. Although these findings derive from a preclinical guinea pig model, they underscore the clinical relevance of identifying effective treatments capable of matching the antitussive efficacy of codeine while avoiding its adverse effects. Importantly, the study reinforces the value of non-opioid drug development and supports the continued use of citric acid in the guinea pig as a reliable and reproducible model for antitussive screening. Given the intrinsic variability of cough responses, future research should incorporate larger sample sizes, include both sexes, and aim to define phenotype-specific therapeutic strategies.

## Data Availability

The raw data supporting the conclusions of this article will be made available by the authors on request.
